# Comparative Risk of Serious Infections Associated With Treatment of Inflammatory Bowel Disease

**DOI:** 10.1093/ibd/izaf218

**Published:** 2025-10-16

**Authors:** Nabeel Khan, Ramaswamy Sundararajan, Dhruvan Patel, Manthankumar Patel, Janak Bahirwani, Nadim Mahmud

**Affiliations:** Department of Gastroenterology, Corporal Michael J. Crescenz VA Medical Center, Philadelphia, PA, United States; Division of Gastroenterology, Perelman School of Medicine, University of Pennsylvania, Philadelphia, PA, United States; Department of Gastroenterology, Corporal Michael J. Crescenz VA Medical Center, Philadelphia, PA, United States; Department of Gastroenterology, Mercy Fitzgerald Hospital, Darby, PA, United States; Department of Gastroenterology, Corporal Michael J. Crescenz VA Medical Center, Philadelphia, PA, United States; Department of Gastroenterology, Kadlec Regional Medical Center, Richland, WA, United States; Department of Gastroenterology, Corporal Michael J. Crescenz VA Medical Center, Philadelphia, PA, United States; Division of Gastroenterology, Perelman School of Medicine, University of Pennsylvania, Philadelphia, PA, United States

**Keywords:** inflammatory bowel disease, ulcerative colitis, Crohn’s disease, infectious disease

## Abstract

**Background:**

There is paucity of data on the risk of infection-related hospitalization among inflammatory bowel disease (IBD) patients exposed to newer biologics. Our aim was to assess the overall and comparative risk of infection-related hospitalization, among IBD patients exposed to different IBD medication classes.

**Methods:**

This was a retrospective cohort study of adult IBD patients in the Veterans Health Administration. New initiators of different IBD medication classes were identified, and the primary outcome was infection requiring hospitalization. Survival analysis methods were used to evaluate the association between time-updated IBD medications and these outcomes, adjusted for key confounders.

**Results:**

The cohort included 14 554 IBD patients. There were 3131 infection hospitalizations over a median follow-up of 49.5 months (interquartile range, 21.2-60.0 months). No other class of medication was associated with a statistically significant difference in the risk of acquiring an infection-related hospitalization compared with patients exposed to a combination therapy of anti-tumor necrosis factor (TNF) inhibitors and thiopurines (TPs). There was a significantly increased risk of gastrointestinal infections with vedolizumab (hazard ration, 1.42; 95% confidence interval, 1.13-1.80; *P = *.003) relative to anti-TNF inhibitors + TPs. Anti-TNF inhibitor monotherapy had a lower risk when compared with vedolizumab.

**Conclusions:**

In this nationwide IBD cohort, there was not an increased risk of infection-related hospitalization among patients exposed to vedolizumab, ustekinumab, and tofacitinib as compared with patients on anti-TNF inhibitor and TP combination therapy and rates of infection-related hospitalization were low overall. These findings should guide clinical decision making and patient-physician discussions when selecting a therapeutic agent with the primary emphasis on efficacy.

## Summary

In this nationwide inflammatory bowel disease cohort, there was not an increased risk of infection-related hospitalization among patients exposed to vedolizumab, ustekinumab, and tofacitinib as compared with patients on anti-tumor necrosis factor inhibitor and thiopurine combination therapy, and rates of infection-related hospitalization were low overall.

Key Messages *What is already known?*

Infection risk influences inflammatory bowel disease treatment decisions, but there is a paucity of comparative real-world data. Most prior studies assess individual biologics with long-term safety across classes remaining understudied.

*What is new here?*

No increased risk of infection-related hospitalization was noted with vedolizumab, ustekinumab, or tofacitinib when compared with anti-tumor necrosis factor inhibitors and thiopurines. Gastrointestinal infections were more common with vedolizumab; overall hospitalization rates were low.

*How can this study help patient care?*

These findings support informed decision making between physicians and patients when selecting inflammatory bowel disease therapies, with greater emphasis on efficacy over infection risk.

## Introduction

The management of inflammatory bowel disease (IBD) has undergone a transformation with the introduction of biologics and small molecules.^1^ Over the past decade, a growing armamentarium of agents with distinct mechanisms of action has been approved, expanding therapeutic options for clinicians.^2^^**,**^^3^ One of the factors that influences this decision is the risk of infection associated with a particular drug. The risk of infection-related hospitalization for individual IBD medications have been evaluated in placebo-controlled trials and follow-up registry studies.^4–9^ However, in the long term, head-to-head comparative data across drug classes with different mechanisms of action remain limited.

To date, only 3 prospective studies have compared the efficacy and safety of biologics: between adalimumab and vedolizumab, between adalimumab and Ustekinumab, and between risankizumab and ustekinumab.^10–12^ Data derived from large healthcare databases have also been utilized to evaluate the risk of infections among IBD patients while comparing one agent with another.^13–15^ While informative, these studies do not offer comprehensive comparative risk estimates for infection-related hospitalization across 5 major therapeutic classes. Similarly, while anti-tumor necrosis factor (TNF) inhibitors have been extensively studied, there is a paucity of real-world data comparing their risk with α4β7 integrin inhibitors, selective interleukin (IL)-12 and IL-23 inhibitors, and JAK1 and JAK3 inhibitors collectively.^7–9^

To address the limitations of prior studies, we conducted a study in a nationwide cohort of IBD patients followed in the Veteran Affairs Healthcare System (VAHS).^16^ Our primary objective was to determine the absolute and relative risk of infection-related hospitalization among IBD patients exposed to 5 different classes of medications. Our secondary aim was to characterize the risk of hospitalization for specific infection types associated with these medication classes.

## Methods

### Study design and cohort creation

This was a retrospective cohort study using data from a well-established Veterans Health Administration cohort of patients with IBD. Derivation of the cohort has been detailed in prior publications.^17^ In the present study, we identified adult patients with established IBD who were newly initiated on 1 of the following medication categories between June 2014 and March 2023: anti-TNF inhibitor biologic therapy alone, anti-TNF inhibitor + thiopurine (TP), vedolizumab, ustekinumab, or tofacitinib. Note that the beginning of the study date range was chosen based on the date of vedolizumab Food and Drug Administration approval, May 20, 2014.^18^ The date of medication category initiation served as the index date for each patient.

### Exposure data

For each patient, we obtained detailed data regarding demographics (age, sex, race), body mass index (BMI), alcohol use, drug abuse, smoking status, and key comorbidities including congestive heart failure, chronic obstructive pulmonary disease (COPD), and diabetes mellitus following previously published methods.^19^^**,**^^20^ IBD type was categorized as ulcerative colitis (UC) or Crohn’s disease (CD), also following prior methods.^19^ The primary exposure in this study was IBD medication based on the categories noted above. Medication data were ascertained using pharmacy tables in the Veterans Health Administration clinical data warehouse, a granular data source that includes outpatient and inpatient medication administrations and pharmacy fill data. Medication data were time updated at 30-day intervals with a 1-month lag applied. For example, if a patient received an anti-TNF inhibitor in a given month, they would be considered exposed for 8 weeks. Additional medication exposure data included corticosteroid (prednisone, prednisolone, dexamethasone, budesonide) and opioid (morphine, hydromorphone, hydrocodone, codeine, oxycodone, fentanyl) use. These medication data (corticosteroids and opioids) were also time updated every 30 days of follow-up.

### Outcome data

The primary outcome was time to hospitalization for any serious infection. Based on a previously published algorithm, infections were ascertained using International Classification of Diseases–Ninth/Tenth Revision hospitalization discharge codes. Categories included lower respiratory tract, skin, gastrointestinal, urinary tract, upper respiratory tract, musculoskeletal, and others (Table S1). Secondary outcomes included hospitalization for specific infection categories.

### Statistical analysis

Descriptive statistics for all major covariate data were presented at the time of first exposure to each IBD medication category, recognizing that patients may contribute to multiple columns if exposed to more than 1 IBD medication category during follow-up. Medians and interquartile ranges (IQRs) and frequencies and percentages were presented for continuous and categorical data, respectively. Incidence rates for any serious infection and individual infection categories were then computed per 1000 person-years, stratified by time-updated IBD medication category exposure. To evaluate the adjusted association between IBD medication category and serious infection hospitalization, unadjusted Kaplan-Meier analysis was initially used, with log-rank testing to identify significant differences in risk by medication category. We then used multivariable Cox regression with data right censored at maximum follow-up or death, and with a maximum time horizon of 60 months. Models were adjusted for a priori variables hypothesized or previously demonstrated to be associated with infectious risk,^21^ including age, sex, race, BMI, IBD type, alcohol use, diabetes mellitus, heart failure, COPD, corticosteroid use, and opioid use. IBD medication category, prednisone use, and opioid use were each treated as time-varying covariates, with data time-updated every 30 days of follow-up in regression models as noted previously. The inclusion of corticosteroids in models served as a proxy for IBD disease flares. Of note, as some patients may have discontinued an IBD medication during follow-up, or discontinued and then switched to another agent, some follow-up time accrued with a “no IBD medication” exposure category. Hazard ratios (HRs) and 95% confidence intervals (CIs) were presented for final regression models, and adjusted survival curves were plotted. To evaluate possible differences in IBD medication impact rated to IBD diagnosis, we also evaluated for an interaction between these variables in the adjusted model. Finally, Cox regression models were fit for hospitalization for each individual infection category, adjusted for the same a priori covariates noted previously. For all hypothesis tests, a 5% alpha threshold was used to determine statistical significance.

### Other considerations

This study received Institutional Review Board approval from the Corporal Michael J. Crescenz Philadelphia Veterans Affairs Medical Center. All data management and analyses were performed using STATA/BE 17.0 (StataCorp).

## Results

### Cohort characteristics

A total of 14 554 patients with IBD were included. During follow-up, a total 13 085 (89.9%) were ever exposed to anti-TNF inhibitor monotherapy, 3914 (26.9%) to anti-TNF inhibitor + TP, 2747 (18.9%) to vedolizumab, 1214 (8.3%) to ustekinumab, and 493 (3.4%) to tofacitinib. Descriptive data at the time of initiation of each IBD medication category are shown in Table 1. Patients initiated on tofacitinib were older at baseline compared with those starting anti-TNF monotherapy (median 55 vs 52 years of age), and tofacitinib patients were also more likely to be on corticosteroids (43.6% vs 20.8%).

**Table 1.** Patient characteristics at first exposure to IBD medications during follow-up.

Factor	**Anti-TNF inhibitor alone** (n** = 13** **085)**	**Anti-TNF inhibitor + TP** (n** = 3914)**	**Vedolizumab** (n** = 2747)**	**Ustekinumab** (n** = 1214)**	**Tofacitinib** (n** = 493)**
**Age, y**	52 (37-65)	50 (37-62)	57 (40-69)	54 (39-67)	55 (38-68)
**Sex**					
** Female**	1609 (12.3)	481 (12.3)	313 (11.4)	165 (13.6)	55 (11.2)
** Male**	11 476 (87.7)	3433 (87.7)	2434 (88.6)	1049 (86.4)	438 (88.8)
**Race**					
** Black**	1860 (14.2)	596 (15.2)	363 (13.2)	180 (14.8)	55 (11.2)
** White**	10 536 (80.5)	3115 (79.6)	2236 (81.4)	986 (81.2)	402 (81.5)
** Other**	689 (5.3)	203 (5.2)	148 (5.4)	48 (4.0)	36 (7.3)
**Alcohol use**	7403 (56.6)	2341 (59.8)	1568 (57.1)	698 (57.5)	275 (55.8)
**Drug abuse**	1273 (9.7)	367 (9.4)	213 (7.8)	108 (8.9)	32 (6.5)
**Smoking status**					
** Current smoker**	442 (3.4)	111 (2.8)	92 (3.3)	22 (1.8)	18 (3.7)
** Never smoker**	5001 (38.2)	1501 (38.3)	1052 (38.3)	443 (36.5)	202 (41.0)
** Past smoker**	3863 (29.5)	1103 (28.2)	978 (35.6)	377 (31.1)	174 (35.3)
** Unknown status**	3779 (28.9)	1199 (30.6)	625 (22.8)	372 (30.6)	99 (20.1)
**Congestive heart failure**	462 (3.5)	110 (2.8)	152 (5.5)	51 (4.2)	22 (4.5)
**Chronic obstructive pulmonary disease**	2334 (17.8)	633 (16.2)	508 (18.5)	219 (18.0)	88 (17.8)
**Diabetes mellitus**	2391 (18.3)	660 (16.9)	546 (19.9)	196 (16.1)	92 (18.7)
**IBD type**					
** Ulcerative colitis**	6019 (46.0)	1689 (43.2)	1480 (53.9)	428 (35.3)	422 (85.6)
** Crohn’s disease**	7066 (54.0)	2225 (56.8)	1267 (46.1)	786 (64.7)	71 (14.4)
**Corticosteroid use**	2728 (20.8)	1058 (27.0)	1013 (36.9)	455 (37.5)	215 (43.6)
**Opioid use**	1644 (12.6)	582 (14.9)	337 (12.3)	181 (14.9)	71 (14.4)

**Values are median (interquartile range) or n (%). P:** atients may be represented in multiple columns if exposed to multiple IBD medication classes during follow-up.

Abbreviations: IBD, inflammatory bowel disease; TNF, tumor necrosis factor; TP, thiopurine.

### Association between IBD medication exposure and any serious infection hospitalization

Over a median follow-up of 49.5 months (IQR, 21.2-60.0 months), a total of 3131 serious infection hospitalizations were observed. The crude incidence rate of this outcome was highest in patients on tofacitinib (6.96 per 1000 person-years; 95% CI, 5.22-9.30), followed by vedolizumab (6.45 per 1000 person-years; 95% CI, 5.76-7.23), and lowest in those on an anti-TNF inhibitor alone (5.25 per 1000 person-years; 95% CI, 5.00-5.50) or not actively exposed to IBD medications (4.43 per 1000 person-years; 95% CI, 4.10-4.79) (Table 2).

**Table 2.** Crude incidence rates of hospitalized infections, overall and by infection class, stratified by time-updated IBD medication exposure.

	Number	Anti-TNF inhibitor alone	Anti-TNF inhibitor + TP	Vedolizumab	Ustekinumab	Tofacitinib	No medications
**Any hospitalized infection**	3131	5.25 (5.00-5.50)	5.84 (5.28-6.47)	6.45 (5.76-7.23)	5.62 (4.19-7.52)	6.96 (5.22-9.30)	4.43 (4.10-4.79)
**Gastrointestinal infection**	1209	1.78 (1.65-1.93)	2.10 (1.78-2.47)	2.84 (2.42-3.34)	2.34 (1.53-3.59)	2.07 (1.25-3.43)	1.54 (1.36-1.74)
**Lower respiratory tract infection**	1082	1.51 (1.39-1.64)	1.47 (1.21-1.79)	2.02 (1.67-2.44)	2.40 (1.60-3.61)	3.11 (2.07-4.69)	1.72 (1.53-1.92)
**Musculoskeletal infection**	72	0.09 (0.06-0.13)	0.14 (0.08-0.26)	0.14 (0.07-0.28)	0.00 (0.00-0.00)	0.13 (0.02-0.91)	0.12 (0.08-0.18)
**Other infection**	618	1.00 (0.91-1.11)	1.09 (0.87-1.37)	0.81 (0.60-1.09)	0.52 (0.22-1.25)	0.91 (0.44-1.92)	0.75 (0.63-0.89)
**Skin infection**	1109	1.68 (1.55-1.82)	1.79 (1.50-2.14)	1.86 (1.53-2.27)	2.56 (1.72-3.82)	2.13 (1.30-3.48)	1.52 (1.35-1.72)
**Upper respiratory tract infection**	294	0.42 (0.36-0.49)	0.50 (0.36-0.69)	0.50 (0.35-0.73)	0.71 (0.34-1.49)	0.39 (0.12-1.20)	0.40 (0.31-0.50)
**Urinary tract infection**	718	0.94 (0.84-1.05)	1.01 (0.80-1.27)	1.51 (1.22-1.88)	1.05 (0.56-1.94)	1.04 (0.52-2.08)	1.23 (1.07-1.40)

**Values are incidence rate (95% confidence interval), unless otherwise indicated.:** All incidence rates are reported per 1000 person-years.

Abbreviations: IBD, inflammatory bowel disease; TNF, tumor necrosis factor; TP, thiopurine.

**Figure 1.**
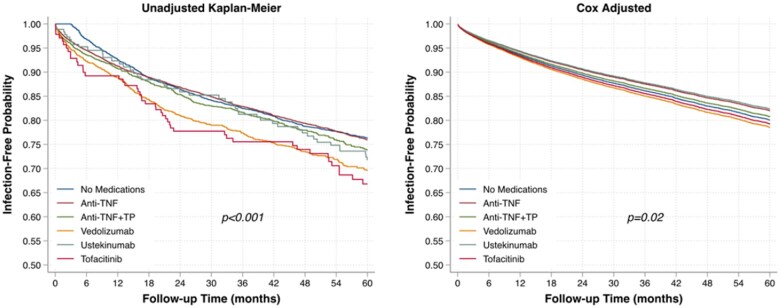
Association between inflammatory bowel disease medication category and hospitalized infection in unadjusted (left) and adjusted (right) survival analysis. TNF, tumor necrosis factor; TP, thiopurine.

Unadjusted Kaplan-Meier analysis demonstrated a significant association between IBD medication category and risk of serious infection hospitalization (log rank *P < *.001) (Figure 1, left). In multivariable Cox regression, there was a significant association between time-updated IBD medication category and serious infection hospitalization (*P = *.02), in which vedolizumab had the highest adjusted risk and ustekinumab had the lowest adjusted risk (Figure 1, right; Table 3, anti-TNF inhibitor + TP as reference group). Adjusted HRs with different IBD medication classes as the reference group are shown in Table S2. Vedolizumab exposure was associated with a higher risk of infection compared with patients on anti-TNF inhibitor monotherapy (Table S2). Variables positively associated with risk of serious infection hospitalization included age, diabetes mellitus, heart failure, COPD, prednisone use, and opioid use (Table 3). There was no significant interaction between IBD medication and IBD diagnosis (*P = *.29).

**Table 3.** Multivariable Cox regression model for serious infection hospitalization.

	Hazard ratio (95% confidence interval)	*P* value
**IBD medication category^a^**		
** No medications**	1.04 (0.91-1.19)	.548
** Anti-TNF inhibitor alone**	0.93 (0.83-1.04)	.198
** Anti-TNF inhibitor + TP**	(ref)	
** Vedolizumab**	1.13 (0.97-1.32)	.124
** Ustekinumab**	0.91 (0.67-1.24)	.544
** Tofacitinib**	1.09 (0.80-1.48)	.593
**Age**	1.01 (1.01-1.01)	<.001
**Sex**		
** Female**	(ref)	
** Male**	0.91 (0.81-1.02)	.120
**Race**		
** Black**	1.08 (0.97-1.20)	.140
** White**	(ref)	
** Other**	1.15 (0.96-1.38)	.126
**Body mass index**	0.98 (0.98-0.99)	<.001
**IBD type**		
** Ulcerative colitis**	(ref)	
** Crohn’s disease**	0.97 (0.90-1.04)	.374
**Alcohol use**	1.40 (1.29-1.51)	<.001
**Diabetes mellitus**	1.25 (1.14-1.37)	<.001
**Heart failure**	1.65 (1.44-1.88)	<.001
**COPD**	1.37 (1.27-1.49)	<.001
**Prednisone use^a^**	2.99 (2.76-3.23)	<.001
**Opioid use^a^**	3.90 (3.62-4.20)	<.001

Abbreviations: COPD, chronic obstructive pulmonary disease; IBD, inflammatory bowel disease; TNF, tumor necrosis factor; TP, thiopurine.

a Time-updated every 30 days of follow-up.

A sensitivity analysis excluding gastrointestinal infections demonstrated largely consistent findings. There was no significant association between IBD medication category and infection-related hospitalization (*P = *.068) (Table S3). A sensitivity analysis among patients ≥65 years of age demonstrated that the same variables remained positively associated with risk of serious infection hospitalization. No significant association between IBD medication category and infection-related hospitalization was observed (*P = *.30) (Table S4).

### Association between IBD medication exposure and hospitalization for individual serious infection categories

The most common individual infection type leading to hospitalization was gastrointestinal infection (1209 events), followed by skin infection (1109 events) and lower respiratory tract infection (1082 events) (Table 2). Vedolizumab had the highest crude incidence rate of gastrointestinal infections at 2.84 per 1000 person-years (95% CI, 2.42-3.34).

Multivariable Cox regression models for each individual infection outcome are shown in Table 4. Notable findings include a significantly increased risk of gastrointestinal infections with vedolizumab (HR, 1.42; 95% CI, 1.13-1.80; *P = *.003) and a significantly increased risk of lower respiratory tract infections with tofacitinib (HR, 1.64; 95% CI, 1.04-2.58; *P = *.035), each relative to anti-TNF inhibitor + TP. Additionally, in-hospital infection-related mortality was significantly associated with specific infection type (*P < *.001); these data are shown in Table S5. For instance, the rates of in-hospital mortality were highest for lower respiratory infections (11.09%) and lowest for musculoskeletal infections (1.39%).

**Table 4.** Multivariable Cox regression models for individual infection classes.

**IBD medication category** ^a^	GI infection	LRTI	MSK	Other	Skin	URTI	UTI
**No medication**	0.96 (0.78-1.19), .703	1.27 (1.00-1.60), .046	0.74 (0.34-1.62), .456	1.15 (0.85-1.55), .358	1.08 (0.86-1.34), .524	0.88 (0.57-1.34), .538	1.47 (1.11-1.95), .007
**Anti-TNF inhibitor alone**	0.89 (0.74-1.07), .225	0.97 (0.79-1.20), .791	0.60 (0.29-1.23), .162	0.98 (0.76-1.26), .887	0.99 (0.81-1.21), .932	0.89 (0.61-1.29), .539	0.92 (0.71-1.19), .535
**Anti-TNF inhibitor + TP**	(ref)	(ref)	(ref)	(ref)	(ref)	(ref)	(ref)
**Vedolizumab**	1.42 (1.13-1.80), .003	1.13 (0.86-1.49), .376	0.86 (0.34-2.22), .760	0.86 (0.59-1.26), .443	1.08 (0.83-1.42), .569	0.99 (0.60-1.65), .983	1.38 (0.99-1.91), .054
**Ustekinumab**	1.12 (0.71-1.77), .630	1.25 (0.79-1.96), .344	Not applicable	0.49 (0.20-1.21), .121	1.38 (0.89-2.15), .148	1.23 (0.54-2.80), .615	0.88 (0.45-1.70), .694
**Tofacitinib**	0.87 (0.51-1.48), .603	1.64 (1.04-2.58), .035	0.59 (0.07-4.67), .617	0.85 (0.39-1.85), .684	1.12 (0.66-1.90), .667	0.67 (0.20-2.19), .505	0.82 (0.39-1.71), .597

**Values are hazard ratio (95% CI), P.:** Each model was adjusted for age, sex, race, body mass index, IBD type, alcohol use, diabetes mellitus, heart failure, COPD, prednisone use (time-updated), and opioid use (time-updated).

Abbreviations: COPD, chronic obstructive pulmonary disease; GI, gastrointestinal; IBD, inflammatory bowel disease; LRTI, lower respiratory tract infection; MSK, musculoskeletal infection; TNF, tumor necrosis factor; TP, thiopurine; URTI, upper respiratory tract infection; UTI, urinary tract infection.

a Time-updated every 30 days of follow-up.

## Discussion

In this nationwide cohort of IBD patients exposed to different classes of immunosuppressive medications, we observed no significant increased risk of infection-related hospitalization across the different medication classes when compared with patients on combination therapy with anti-TNF inhibitor agents and TPs. Consistent with prior literature, well-established risk factors like age, opioid use, and prednisone use were associated with an increased risk.

Despite the approval of novel therapies for IBD, data on the comparative risk of infections associated with different classes of IBD medications remains sparse. Only 3 head-to-head trials are available for comparison: the VARSITY (An Efficacy and Safety Study of Vedolizumab Intravenous [IV] Compared to Adalimumab Subcutaneous [SC] in Participants With Ulcerative Colitis; NCT02497469; EudraCT 2015-000939-33) trial, which compared treatment differences between vedolizumab and adalimumab in UC patients; SEAVUE (Safety and Efficacy of Adalimumab Versus Ustekinumab for One Year; NCT03464136) trial, which compared treatment differences between ustekinumab and adalimumab in CD patients; and the SEQUENCE (Study Comparing Intravenous (IV)/​Subcutaneous (SC) Risankizumab to IV/​SC Ustekinumab to Assess Change in Crohn's Disease Activity Index (CDAI) in Adult Participants With Moderate to Severe Crohn's Disease; NCT04524611) trial, which compared treatment differences between risankizumab and ustekinumab in patients with CD.^10–12^ All these trials, while methodologically rigorous, had limited duration of follow-up and few serious infection events. For instance, the VARSITY trial reported only 15 serious infections, of which 8 were on adalimumab, and there were 9 serious infections in the SEAVUE trial, of which 5 were on adalimumab. The SEQUENCE trial had 19 infections, of which 8 were on risankizumab and 11 were on ustekinumab. These small numbers prevented any statistical analysis and provided limited information.

Prior real-world studies have compared one drug to another rather than evaluating all major therapeutic classes together. Cheng et al^15^ compared the risk of infections associated with ustekinumab and tofacitinib and compared them with anti-TNF inhibitor agents, but the duration of exposure to both tofacitinib and ustekinumab was about 6 months as compared with a median follow-up of 50 months in our study. Solitano et al,^22^ through a comprehensive meta-analysis of studies that compared vedolizumab and ustekinumab with each other and to anti-TNF inhibitors, observed varied results depending upon the phenotype of IBD as well. Kirchgesner et al,^13^ using a French National Health Insurance database, found the incidence of infection requiring hospitalization to be 10.5, 18.9, and 22.4 per 1000 person-years in those exposed to thiopurine monotherapy, anti-TNF inhibitor monotherapy, and combination therapy, respectively. The unique aspect of our study is that it expands upon this work by providing comparative data across 5 medication classes.

It has been postulated that as ustekinumab selectively targets the common p40 subunit shared by IL-12 and IL-23 and inhibits the receptors for these cytokines on T cells, natural killer cells, and antigen-presenting cells in contrast to the broader immunosuppressive effects of anti-TNF inhibitor agents, it should have a more favorable safety profile.^23^^**,**^^24^ Similarly the gut-selective mechanism of action of vedolizumab has the potential to improve its safety profile compared with other agents.^25^ In our analysis, when comparing the combination of TP and anti-TNF agents, as it has been shown to be more effective than IFX or TP monotherapy,^26^^**,**^^27^ we found that no other class of medication was associated with a statistically significant difference in the risk of acquiring an infection associated with hospitalization. The crude incidence rates of infection-related hospitalizations were also similar ranging from 5.25 to 6.96 per 1000 person-years, with the lowest rates associated with anti-TNF monotherapy and the highest with tofacitinib. In adjusted analyses, using anti-TNF monotherapy as the reference drug, vedolizumab exposure was associated with a higher risk of infections. This is an important finding, as today there are multiple agents to choose from, and physicians and patients are concerned about the risk of infections when choosing an agent. Our data suggest that while choosing an agent, more emphasis should be placed on efficacy, especially in the context of relative comparable safety. Furthermore, the overall risk of acquiring an infection associated with hospitalization was low across the spectrum of medications highlighting the safety of these drugs in an older population. The most frequently observed infection was those related to the gastrointestinal tract, followed by skin and infections of the lower respiratory tract. While comparing individual infections, it was found that vedolizumab was associated with an increased risk of gastrointestinal infections, which has been observed in other studies as well.^28^^**,**^^29^ In a sensitivity analysis that explored the risk of infection-related hospitalizations while excluding gastrointestinal infections, the overall findings remained largely consistent. However, we did not observe a significant association between IBD medication category and infection-related hospitalization. This suggests that gastrointestinal infections were an important driver of differential infectious risk by medication category. Tofacitinib was associated with an increased risk of lower respiratory tract infections, a finding which warrants further investigation. The incidence rates for hospitalizations with each individual infection were quite similar among the different medication classes.

In contrast to medications, our study observed that other host-related factors were associated with an increased risk of infections. Age has been shown to be a leading risk factors for infections among the IBD patient population.^30–32^ Our study also reinforces this, with age being a statistically significant risk factor for infection-related hospitalization (HR, 1.01; 95% CI, 1.01-1.01, *P *≤ .001). Additionally, when evaluating patients 65 years of age and older in a sensitivity analysis, previously established risk factors like corticosteroid use remain significant. However, the overall impact of IBD medication category was not significant, which could be the result of smaller population subgroups, limiting our ability to identify any meaningful differences. Furthermore, we demonstrated a significantly higher risk of infection related hospitalization for patients on prednisone and opioids and those having COPD, as has been previously reported.^19^ These findings give further credence to the utilization of steroid-sparing drugs in the management of IBD.^8^ Interestingly we found that an increase in BMI is associated with a significantly lower risk of infection hospitalization (HR, 0.98; 95%CI, 0.98-0.99; *P < *.001). These findings somewhat align with prior work in which the use of biologics among IBD patients was not associated with an increased risk of infections.^33^ These findings underscore the value of individualized risk stratification and the potential value of steroid-sparing therapies in routine clinical practice.

Our study has many strengths. We utilized a large nationwide cohort of IBD patients who receive care within the VAHS, which caters to over 9 million veterans annually.^16^ We utilized the centralized Veterans Affairs pharmacy records to gather data on medications, which ensured that even if patients changed locations their medication exposure would be captured. The median age among all groups of medications was above 50 years, and as this elderly group is the most at risk of infection-associated complications, our study captured this vulnerable population. However, this study is not without its limitations. Because the Veterans Affairs cohort predominantly includes older white males, this limits the generalizability of the study. We did not include the newer IL-23 agents (mirikizumab, risankizumab, and guselkumab) due to insufficient patient numbers. However, the SEQUENCE trial, while limited in its follow-up, did not show any difference in serious infections rates between the IL-12/23 and the IL-23 agents. We also did not include the S1P (sphingosine-1 phosphate) agents (ozanimod and etrasimod) due to a limited number of patients receiving this medication. The retrospective design and the inability to track serious infections occurring outside the Veterans Affairs system are inherent limitations as well that may lead to outcomes misclassification. However, as these patients were receiving their medications in the Veterans Affairs and being actively followed in the system, outcomes misclassification from outside hospitalizations are likely to be minimal. We did not restrict the International Classification of Diseases–Tenth Revision code to the primary position when identifying infection-related hospitalizations. While this approach was utilized to identify all clinically relevant infection-related hospitalizations, it may have included events where infection was a contributing factor, as opposed to being the primary reason for hospital admission. Finally, although we aimed to comprehensively account for plausible confounders and incorporated key time-updating covariates and medication exposures in models, there remains the potential for residual confounding.

In conclusion, within this large national VAHS IBD cohort, no increase in infection-related hospitalization was observed among patients exposed to anti-TNF inhibitor, vedolizumab, ustekinumab, and tofacitinib as compared with patients on combination anti-TNF inhibitor and TP therapy. The overall rates of infection associated with hospitalizations across all medication groups were low. These findings highlight the safety of these steroid-sparing agents and may help guide clinical decision making and patient-physician discussions, with the primary emphasis being on efficacy when choosing a therapeutic agent, in the context of comparable safety profiles of these therapies.

## Supplementary Material

izaf218_Supplementary_Data

## Data Availability

The data for this manuscript cannot be made available in accordance with the Health Information Portability and Accountability Act rules. However, de-identified data (without patient name and Social Security number), can be made available upon reasonable request.
