# Supplementary material for: Comparative Risk of Serious Infections Associated With Treatment of Inflammatory Bowel Disease
Source: Inflamm Bowel Dis. 2025 Oct 16;32(2):199–206. doi: 10.1093/ibd/izaf218 (PMC12857427; doi:10.1093/ibd/izaf218)
Supplement: izaf218_Supplementary_Data [file izaf218_supplementary_data.zip › Supplemental data.docx]

**Supplemental Table 1 - Classification of Infection Categories with ICD-10 and ICD-9 Codes**

| **Infection Category** | **ICD-10** | **ICD-9** |
| --- | --- | --- |
| Lower respiratory tract infections | A48.1; B01.2; B05.2; B25.0; J10-J18 | 052.1; 055.2; 480; 481; 482.0; 482.1; 482.2; 482.3; 482.4; 482.81; 482.82; 482.83; 482.84; 482.89; 482.9; 483.0; 483.1; 483.8; 484.1; 484.7; 484.8; 485; 486; 487; 488 |
|  | A37; A42.0; B39-B40; B44; B48.5; B58.3; B59; B95.3; J20-J22; U04 | 033; 039.1; 041.2; 115; 116.0; 117.3; 130.4; 466; 484.3; 484.6 |
|  | J85 | 513 |
|  | J86 | 510 |
| Upper respiratory tract infections | H70 | 383.0 |
|  | A36.1 | 032.1; 032.2 |
|  | J01 | 461 |
|  | J02 | 034.0 |
|  | J36; J39.0-J39.1 | 032.1; 475; 478.21; 478.22; 478.24; 478.71 |
|  | A36.0; J03 | 032.0; 463 |
|  | A36.2; J04-J05 | 032.3; 464; 465; 572.0 |
|  | A36.8-A36.9; J06 | 032.81; 032.82; 032.83; 032.84; 0.32.9; 465 |
|  | H60.0-H60.3; H65.1; H66; H68.0 | 380.1; 382.0 |
| GI infections | A00-A08 | 001-009; 040.4 |
|  | B15; B17; B25.1 | 070.0; 070.1; 070.20; 070.21; 070.30; 070.31; 070.41-070.43; 070.51; 070.52; 070.53 |
|  | K80-K810; K830, K87.00; B25.8 | 574.0; 574.3; 574.6; 575.0; 576.1; 576.1 |
|  | K750 | 572.0 |
|  | B00.8; K23.80 | 054.6; 054.7 |
| Skin and subcutaneous tissue infections | A46 | 035 |
|  | B35-B36 | 110-111 |
|  | L02-L03 | 680-682 |
|  | B00-B02; B05-B06; B08-B09; A60; | 052.2; 052.8; 052.9; 053.2; 053.7; 053.8; 053.9; 054.1; 054.2; 054.5; 054.8; 054.9 |
|  | A36.3; K11.3-K12.2; L00-L01;L04-L05; L08; L30.3; M72.6 | 032.85; 527.3; 528.3; 683; 684; 685; 686; 695.81; 728.86 |
| Urinary tract infections | N10 | 590 |
|  | N41.0; N41.2; N41.3 | 601.0-601.3 |
|  | N30.0 | 595.0 |
|  | N70.0 | 614.0 |
|  | A50-A53; I98.0 | 090-097 |
|  | A54 | 098 |
|  | A55-A56 | 099.1 |
|  | N45 | 604 |
|  | N39.0; N73.3; N77.1 | 599.0; 614.3; 614.5 |
| Musculoskeletal infections | M00-M01 | 711.0; 711.1; 711.4-711.9 |
|  | M60.0 | 728.0 |
| Other infections | B00.5; B30; H00; H03.1; H06.1; H13.1; H19.1-H19.2 | 054.4; 077; 373.1; 373.2 |
|  | A32.1; A39; A80-A89; B00.3-4; B01.0; B02.1; B05.1; B06.0; G00-G02; G04-G07 | 027.0; 036; 045-049; 052.0; 052.7; 053.0; 053.1; 054.3; 055.0; 055.1; 056.0; 062-064; 071; 320; 321.0-321.3; 323.0-323.2; 323.4; 324.0; 324.1; 324.9 |
|  | A02.1; A32.7; A39.2; A39.4; A39.8; A39.9; A40-A41; B37.7; R57.2; R65.0-R65.1 | 614.0; 027.0; 038.0; 038.1; 038.2; 038.3; 038.4; 038.8; 995.90; 995.91 |
|  | A20-A28; A32; A34-A35; A38; A42-A44; A48.0; A48.2-A49.9; B95-B97 | 020-026; 027.0-027.2; 027.8; 027.9; 034.1; 037; 038.3; 039.0; 039.2; 039.3; 039.8; 039.9; 040.0; 040.3; 040.81; 040.82; 040.89; 041.0; 041.1; 041.3; 041.4; 041.5-041.7; 041.81-041.85 |
|  | A65-A69 | 087; 101; 102; 103; 104.0 ; 104.8; 104.9 |
|  | A75-A79 | 080-083 |
|  | A90-A99; B25-B27; B33-B34 | 055.7-055.9; 056.7-056.9; 057-061; 064-066; 072; 074.0-074.3; 074.8; 075; 078.2; 078.4; 078.5; 078.5; 078.5; 078.6; 078.7; 078.8; 079 |
|  | B37-B49; B59 | 039.4; 112; 114; 116.1; 116.2; 117.0-117.2; 117.4-117.9; 118 |
|  | A07.2; B50-B83 | 084-086; 088.82; 088.89; 120-122; 123.0-123.4; 123.5; 123.6; 123.8; 123.9; 124; 125.0-125.7; 125.9; 126; 127.0-127.9; 128; 130.0; 130.3; 130.5; 130.7-130.9 |
|  | B99.9 | 136.9 |
|  | I30.1; I33.0 | 421.0; 421.1 |
|  | A15-A19; A31; K23.0; K67.3; K93.0; M01.1; M49.0; M90.0; N33.0; N74.0; N74.1 | 010-018; 031 |

**Supplemental Table 2 - Multivariable Cox Regression Results for Serious Infection Hospitalization with Vedolizumab or Ustekinumab used as Reference Groups for the IBD Medication Category Variable***

|  | Hazard Ratio  (95% Confidence Interval) | P-value | Hazard Ratio  (95% Confidence Interval) | P-value | Hazard Ratio  (95% Confidence Interval) | P-value |
| --- | --- | --- | --- | --- | --- | --- |
| IBD Medication Category*** |  |  |  |  |  |  |
| No Medications | 0.92 (0.80, 1.06) | 0.258 | 1.15 (0.85, 1.55) | 0.377 | 1.12 (1.02, 1.24) | 0.019 |
| Anti-TNF Alone | 0.82 (0.72, 0.93) | 0.002 | 1.02 (0.76, 1.38) | 0.885 | (ref) | — |
| Anti-TNF + TP | 0.88 (0.76, 1.03) | 0.124 | 1.10 (0.81, 1.50 | 0.544 | 1.08 (0.96, 1.21) | 0.198 |
| Vedolizumab | (ref) |  | 1.24 (0.91, 1.70) | 0.174 | 1.22 (1.07, 1.38) | 0.002 |
| Ustekinumab | 0.80 (0.59, 1.10) | 0.174 | (ref) | 0.544 | 0.98 (0.73, 1.32) | 0.885 |
| Tofacitinib | 0.96 (0.70, 1.31) | 0.811 | 1.20 (0.79, 1.81) | 0.392 | 1.17 (0.87, 1.57) | 0.292 |

**** Model covariates are otherwise the same as those shown in Table 3***

**Supplemental Table 3 – Sensitivity Analysis Excluding GI Infections**

|  | Hazard Ratio  (95% Confidence Interval) | P-value |
| --- | --- | --- |
| IBD Medication Category***† |  |  |
| No Medications | 1.09 (0.94, 1.27) | 0.234 |
| Anti-TNF Alone | 0.94 (0.83, 1.07) | 0.352 |
| Anti-TNF + TP | (ref) |  |
| Vedolizumab | 1.04 (0.87, 1.24) | 0.699 |
| Ustekinumab | 0.99 (0.72, 1.37) | 0.954 |
| Tofacitinib | 1.22 (0.88, 1.69) | 0.228 |
| Age | 1.02 (1.01, 1.02) | <0.001 |
| Sex |  |  |
| Female | (ref) |  |
| Male | 0.87 (0.77, 0.98) | 0.028 |
| Race |  |  |
| White | (ref) |  |
| Black | 1.08 (0.96, 1.21) | 0.200 |
| Other | 1.01 (0.81, 1.26) | 0.919 |
| Body Mass Index | 0.99 (0.98, 0.99) | <0.001 |
| IBD Type |  |  |
| Ulcerative Colitis | (ref) |  |
| Crohn’s Disease | 1.04 (0.96, 1.13) | 0.347 |
| Alcohol Use | 1.42 (1.30, 1.54) | <0.001 |
| Diabetes Mellitus | 1.32 (1.20, 1.45) | <0.001 |
| Heart Failure | 1.69 (1.47, 1.95) | <0.001 |
| COPD | 1.47 (1.34, 1.61) | <0.001 |
| Prednisone Use* | 2.87 (2.63, 3.13) | <0.001 |
| Opioid Use* | 3.95 (3.64, 4.29) | <0.001 |

**** Time updated every 30-days of follow-up.
† Joint p-value=0.0681***

**Supplemental Table 4 – Sensitivity Analysis Among Patients Age ≥65 Years**

|  | Hazard Ratio  (95% Confidence Interval) | P-value |
| --- | --- | --- |
| IBD Medication Category*† |  |  |
| No Medications | 1.18 (0.93, 1.50) | 0.167 |
| Anti-TNF Alone | 1.03 (0.84, 1.27) | 0.775 |
| Anti-TNF + TP | (ref) |  |
| Vedolizumab | 1.15 (0.89, 1.50) | 0.280 |
| Ustekinumab | 0.79 (0.47, 1.32) | 0.360 |
| Tofacitinib | 1.18 (0.75, 1.85) | 0.469 |
| Age | 1.03 (1.02, 1.04) | <0.001 |
| Sex |  |  |
| Female | (ref) |  |
| Male | 0.70 (0.52, 0.95) | 0.022 |
| Race |  |  |
| White | (ref) |  |
| Black | 1.05 (0.85, 1.29) | 0.661 |
| Other | 0.72 (0.42, 1.24) | 0.239 |
| Body Mass Index | 0.99 (0.98, 1.00) | 0.014 |
| IBD Type |  |  |
| Ulcerative Colitis | (ref) |  |
| Crohn’s Disease | 1.00 (0.89, 1.12) | 0.970 |
| Alcohol Use | 1.35 (1.20, 1.52) | <0.001 |
| Diabetes Mellitus | 1.27 (1.13, 1.44) | <0.001 |
| Heart Failure | 1.76 (1.49, 2.07) | <0.001 |
| COPD | 1.47 (1.30, 1.65) | <0.001 |
| Prednisone Use* | 2.84 (2.51, 3.22) | <0.001 |
| Opioid Use* | 3.12 (2.76, 3.54) | <0.001 |

**** Time updated every 30-days of follow-up.
† Joint p-value=0.3002***

**Supplemental Table 5 – Hospital Infection-Related Mortality by Infection Type***

| Infection Type | Hospital Mortality | | Total |
| --- | --- | --- | --- |
|  | Yes | No |  |
| GI | 37 (3.06%) | 1,172 (96.94%) | 1,209 |
| LRTI | 120 (11.09%) | 962 (88.91%) | 1,082 |
| MSK | 1 (1.39%) | 71 (98.61%) | 72 |
| Other | 34 (5.50%) | 584 (94.50%) | 618 |
| Skin | 33 (2.98%) | 1,076 (97.02%) | 1,109 |
| Upper Resp | 8 (2.72%) | 286 (97.28%) | 294 |
| UTI | 36 (5.01%) | 682 (94.99%) | 718 |

**** Fisher’s Exact p<0.001***
